# Supplementary material for: Comparison of model feature importance statistics to identify covariates that contribute most to model accuracy in prediction of insomnia
Source: PLoS One. 2024 Jul 2;19(7):e0306359. doi: 10.1371/journal.pone.0306359 (PMC11218970; doi:10.1371/journal.pone.0306359)
Supplement: Attachment — Submitted filename: 230918 Response to Reviewers.docx [file pone.0306359.s001.docx]

Reviewers' comments:

Reviewer's Responses to Questions

**Comments to the Author**

1. Is the manuscript technically sound, and do the data support the conclusions?

The manuscript must describe a technically sound piece of scientific research with data that supports the conclusions. Experiments must have been conducted rigorously, with appropriate controls, replication, and sample sizes. The conclusions must be drawn appropriately based on the data presented.

Reviewer #1: Yes

Reviewer #2: Yes

2. Has the statistical analysis been performed appropriately and rigorously?

Reviewer #1: Yes

Reviewer #2: Yes

3. Have the authors made all data underlying the findings in their manuscript fully available?

The [PLOS Data policy](http://www.plosone.org/static/policies.action#sharing) requires authors to make all data underlying the findings described in their manuscript fully available without restriction, with rare exception (please refer to the Data Availability Statement in the manuscript PDF file). The data should be provided as part of the manuscript or its supporting information, or deposited to a public repository. For example, in addition to summary statistics, the data points behind means, medians and variance measures should be available. If there are restrictions on publicly sharing data—e.g. participant privacy or use of data from a third party—those must be specified.

Reviewer #1: Yes

Reviewer #2: Yes

4. Is the manuscript presented in an intelligible fashion and written in standard English?

PLOS ONE does not copyedit accepted manuscripts, so the language in submitted articles must be clear, correct, and unambiguous. Any typographical or grammatical errors should be corrected at revision, so please note any specific errors here.

Reviewer #1: Yes

Reviewer #2: Yes

5. Review Comments to the Author

Please use the space provided to explain your answers to the questions above. You may also include additional comments for the author, including concerns about dual publication, research ethics, or publication ethics. (Please upload your review as an attachment if it exceeds 20,000 characters)

Reviewer #1: Additional comments for the author:

1. The title, Comparison of model feature importance statistics to identify the covariates that most contribute to model accuracy, doesn’t tell in what situation the comparison was made, please include the information that it was to insomnia data.
2. The Dataset and Cohort Selection section should inform the sample size.
3. Methods section can also include data analysis methods, such as definitions or a brief description of machine learning models and model gain statistics.
4. The confusion matrix (Figure 1, line 203) would not be the correlation matrix ? A confusion matrix table reports the absolute frequency of true positives, false negatives, false positives, and true negatives.
5. Selecting a better model that identifies the most relevant risk factors requires not only good model fit parameters but also smaller errors. Residual measures should also be considered for comparison models (such as MAE, MSE, MAPE,...).

Reviewer #2: The article identifies features that best predict insomnia using a machine learning model. Based on the information provided, I think the paper overall was solid methodologically, although a few things can be clarified. I have a few suggestions and comments:

1. I think my major confusion is it is unclear if the aim of the paper is methodological or if it’s trying to contribute to the insomnia literature. If the goal is content, the title will need to be changed to reflect that. If the goal is more of a methodological contribution, then probably the introduction needs to be reworked, you will want to start with the contributions of machine learning models and then discuss how you’re going to use this paper to illustrate this point, such as in insomnia research. There are two different aims here: Line 77-82, as well as Line 89-92. Of course, you can still contribute to the literature of insomnia research while being a methods paper, but the focus is unclear.

2. It is unclear what software the authors used to run the analysis, so it is difficult to assess whether things were done properly. Is it Phyton, R, or something else? Please provide citations for gain, cover, and Shapley values and which packages were used to perform these. If you calculated them from scratch, then provide the GitHub link or codes to ensure reproducibility, which is the policy of PLOSOne. I’m less familiar with this approach, I’m most familiar with the agnostic method of variable ranking (perhaps cite the book Interpretable Machine learning). I know Greenwell’s R package to do this, not sure what was used in this analysis. Based on your description, your way of variable importance makes sense, but I’d like to see more citation to see what your contributions are and if this method has been used before, or you wrote new packages.

Greenwell B.M., Boehmke B.C., McCarthy A.J. A Simple and Effective Model-Based Variable Importance Measure. arXiv. 20181805.04755
Molnar, C. (2022). Interpretable Machine Learning: A Guide for Making Black Box Models Explainable (2nd ed.).[christophm.github.io/interpretable-ml-book/](http://christophm.github.io/interpretable-ml-book/)

3. Related to above, this comes back to my confusion whether the aim is to suggest other researchers to focus more on ML when identifying the most important predictors instead of using multivariable regression, or if you simply want to contribute to insomnia literature using ML. If the former, please state how other researchers can use the technique in their work. A few other methods papers have been written on variable importance, such as the one below. This paper has a step by step of how to identify the most important predictors when running a descriptive epidemiological analysis, which is what this is. Again, not sure if that’s the aim, if you want to encourage other researchers to apply ML instead of multivariable regression. I’d recommend looking into this work; they also discussed what you put under discussion about interaction terms and the challenge when different models chose inconsistent variables as the most important predictors. I believe this is an important future direction that should be continually discussed.
Dharma C, Fu R, Chaiton M. Table 2 Fallacy in Descriptive Epidemiology: Bringing Machine Learning to the Table. Int J Environ Res Public Health. 2023 Jun 21;20(13):6194. doi: 10.3390/ijerph20136194. PMID: 37444042; PMCID: PMC10340623.

4. I do not understand why the univariable filter was first done before the machine learning models, I thought one of the benefits of ML is to indeed filter out the unnecessary variables. Again, I will need to see citations for this or explain why this was done. The theme is, there is a lack of reliable citation for how the methods were performed and why certain decisions were made.

5. Minor: Unless I misunderstood, “Legend” caption under Table 3 should have gone under Figure 1, otherwise the description does not make sense about the y-axis and x-axis in Table 3.

6. PLOS authors have the option to publish the peer review history of their article ([what does this mean?](https://journals.plos.org/plosone/s/editorial-and-peer-review-process#loc-peer-review-history)). If published, this will include your full peer review and any attached files.


If you choose “no”, your identity will remain anonymous but your review may still be made public.


**Do you want your identity to be public for this peer review?** For information about this choice, including consent withdrawal, please see our [Privacy Policy](https://www.plos.org/privacy-policy).

Reviewer #1: No

Reviewer #2: No
