# Supplementary material for: Comparison of model feature importance statistics to identify covariates that contribute most to model accuracy in prediction of insomnia
Source: PLoS One. 2024 Jul 2;19(7):e0306359. doi: 10.1371/journal.pone.0306359 (PMC11218970; doi:10.1371/journal.pone.0306359)
Supplement: Attachment — Submitted filename: Response to Reviewers.docx [file pone.0306359.s002.docx]

**We would like to extend our sincere gratitude to the reviewers for their insightful comments and constructive criticisms. Their detailed feedback has been invaluable in guiding our revisions, allowing us to address critical aspects of our methodology and analysis that required further clarification and enhancement. The reviewers’ expertise and thoughtful suggestions have significantly contributed to the depth and rigor of our study, ensuring a more comprehensive and robust examination of the predictive models utilized in the context of insomnia prediction. Their contributions have not only facilitated methodological improvements but also enriched our discussion, leading to a more nuanced understanding of the complexities involved in variable selection within machine learning and regression models. We are grateful for the opportunity to refine our work through this collaborative and iterative process, which has undeniably strengthened the quality and impact of our research. We have responded to all reviewer feedback to the best of our abilities.**

Reviewer Comments Line by Line Response:

Please use the space provided to explain your answers to the questions above. You may also include additional comments for the author, including concerns about dual publication, research ethics, or publication ethics. (Please upload your review as an attachment if it exceeds 20,000 characters)

Reviewer #1: 1. (Major) The authors emphasize the importance of comparing how various model metrics rank covariates due to the critical role of sleep in an individual's physical and mental health. They applied four different machine learning models to an insomnia dataset and found that these models provided additional information into the ranking of covariates for predicting insomnia compared to regression models based on the fact that machine learning models take into account the existing collinearity between covariates when ranking the important ones, as stated in the “Discussion” (p 263 – 265): “ Therefore, we argue that machine learning brings a new way of evaluating variables beyond traditional regression, as it can account for confounding variables and identify important variables for future studies."

However, variable selection metrics, such as gain in machine learning, generally cannot directly account for confounding variable or evaluate the existing correlations or collinearities among covariates. Gain is typically calculated based on a feature's ability to reduce impurity or enhance model accuracy. The collinearity among covariates can indirectly impact the variable selection process. In the presence of high correlations, the gain of one variable may be dampened by another correlated one, leading to an underestimation of its true importance. Therefore, correlation analysis should be performed before the selection of covariates or considering other prior analyses, such as variable normalization or standardization, and principal component analysis.

Another way to ensure that machine learning models added information to the covariate selection ranking is for both models to exhibit similar performance. However, there are no performance evaluation metrics for regression models for comparison. For an assessment of which model provides the best prediction of insomnia based on covariates, in addition to observing the performance of both models' fits, it would be necessary to evaluate the residuals—the difference between the observed insomnia probability in the data and the insomnia probability estimated by the models, which can be obtained through metrics such as MAE, MSE, MAPE, ….

Although the authors establish well in “Introduction” (p 74): “While traditional statistical models focus on hypothesis testing and estimation, machine learning models aim to predict outcomes by learning patterns in the data.”, the conclusion of the study is only that the machine learning and regression models selected different covariates considering the order of importance in predicting the probability of insomnia, and there was no relationship between the ranking of important variables associated with sleep disorder of the models.

Since the selection of predictor variables for insomnia may differ between the models, it is important to carefully consider the performance of the models and the predicted values of both machine learning and regression models for a comparison between them. Therefore:

1. The discussion will need to be revised.

Overall Response: Thank you for these comments, we have revised the manuscript accordingly.

In our exploration of the predictive modeling of insomnia using both traditional statistical and machine learning approaches, we identified several methodological and analytical challenges that highlight the need for ongoing research in this field. The application of machine learning models to insomnia datasets revealed the potential for these models to provide a nuanced understanding of covariate importance, taking into account the collinearity among variables. This stands in contrast to traditional regression models, which may not fully account for such complexities. However, the reliance on variable selection metrics in machine learning, such as gain, necessitates a careful consideration of their limitations, particularly regarding the indirect treatment of confounding variables and collinearities.

Our analysis underscores the importance of incorporating additional statistical methods, such as correlation analysis, variable normalization, or principal component analysis, prior to the selection of covariates. These methods could mitigate the effects of collinearity and provide a more accurate assessment of variable importance. Furthermore, the comparison between machine learning and regression models in our study was primarily qualitative, based on the ranking of covariates. To strengthen this comparison, a quantitative assessment involving performance metrics and a residuals analysis would be invaluable. Such an analysis would offer a clearer picture of the predictive accuracy of each model type and the reliability of their variable importance rankings.

Moreover, our study highlights the gap in the literature regarding the comprehensive evaluation of insomnia predictors using machine learning techniques. While traditional statistical models have predominantly focused on hypothesis testing and estimation, machine learning models present an opportunity to predict outcomes by identifying complex patterns in the data. This distinction suggests that machine learning could complement traditional approaches by uncovering previously unknown interactions and predictors of insomnia. Yet, the explicit comparison of these models' performance and the detailed examination of their predictive capabilities remain areas ripe for further investigation.

In addition, the documentation of our dataset and cohort selection process revealed the necessity for greater transparency in reporting research methodologies. Specifically, providing detailed information about the sample size and selection criteria is essential for ensuring the reproducibility and validity of research findings.

In conclusion, our study contributes to the growing body of research on insomnia prediction by highlighting the potential synergies between machine learning and traditional statistical models. However, it also emphasizes the need for methodological enhancements and deeper analytical rigor to fully leverage the strengths of each modeling approach. Future research should focus on addressing these limitations through the integration of additional statistical techniques, comprehensive model performance evaluations, and clearer articulation of research contributions within the context of existing literature.

2. (Minor) In the "Dataset and Cohort Selection" section, lines 112 and 113 state: "... All patients in the dataset with full insomnia data were included in this study." The question posed in the initial review regarding the number of patients included was not addressed (The Dataset and Cohort Selection section should inform the sample size).

We addressed this minor concern in the paper in the methods section!

Reviewer #2: Thank you for the opportunity to rereview this paper. At first I thought I received the wrong version since the authors did not do the typical point by point response to the points raised by the reviewers, but I see now that the revisions have been made. The authors have sufficiently addressed all the comments I raised.

We thank the reviewer for the comments
